# Bioinformatics analysis and experimental validation of the potential relationship between bacterial lipopolysaccharide and oral squamous cell carcinoma

**DOI:** 10.1371/journal.pone.0329231

**Published:** 2025-08-21

**Authors:** Wannan Gao, Hongyan Yuan, Song Qing

**Affiliations:** 1 College of Stomatology, North Sichuan Medical College, Affiliated Hospital of North Sichuan Medical College, Nanchong, China; 2 College of Stomatology, Chongqing Medical University, Chongqing, China; Brigham and Women's Hospital Division of Cardiovascular Medicine, INDIA

## Abstract

**Background:**

Advances in science and medicine have led to the identification of bacterial virulence factors (including lipopolysaccharide, LPS) and their key role in the occurrence and outcome of tumors. However, the effect of LPS on oral squamous cell carcinoma (OSCC) has yet to be fully understood.

**Objective:**

Hence, based on host genes related to bacterial LPS, the study investigated the potential role and mechanism of oral bacteria in OSCC via bioinformatics analysis and experimental validation.

**Methods:**

The sequencing datasets of OSCC were screened using the GEO database and the bacterial LPS-related genes were searched in the GeneCards database to identify the LPS-related differentially expressed genes (LR-DEGs) in OSCC. The molecular mechanism of bacteria affecting OSCC was explored through GO and KEGG enrichment analysis, as well as protein-protein interaction (PPI) network and module analysis. Subsequently, seven algorithms were integrated to identify the LPS-related hub genes (LRHGs), and their diagnostic specificities were explored by receiver operating characteristic (ROC) and transcription levels were verified by qRT-PCR. Immune infiltration was then analyzed.

**Results:**

We found a total of 345 LR-DEGs. GO and KEGG enrichment analysis demonstrated that the LR-DEGs were mainly enriched in inflammation-related pathways including cytokine-cytokine receptor interaction and IL-17 signaling, suggesting that bacteria may promote the development of OSCC through LPS-related gene-mediated inflammatory response. PPI and module analysis results revealed the presence of a complex regulatory network involving LR-DEGs. Totally, five LRHGs (including *Cxcl8*, *Cxcl10*, **Il-1*β*, *Il-6* and *Mmp9*) were screened out. Based on ROC analysis, the five LRHGs represented potential diagnostic biomarkers for OSCC (AUC > 0.7). The results of qRT-PCR, WB, ELISA and IF indicated that all LRHGs were upregulated in OSCC (P < 0.05). Immune infiltration analysis showed that LRHGs were closely related to the immunocyte infiltration level, suggesting a potential target for OSCC immunotherapy. In this study, 345 LR-DEGs and 5 LRHGs were identified in bacterial LPS-regulated OSCC progression. Importantly, the 5 LRHGs may mediate the OSCC progression in the host through inflammation-related pathways. These findings suggest that bacterial LPS plays a vital role in OSCC.

**Conclusion:**

Our study provides novel insights into the pathogenesis and development of oral bacteria in OSCC. The LRHGs identified in this study are crucial for the diagnosis of OSCC, and also provide new insights into the molecular mechanisms and targeted therapies of OSCC.

## 1. Introduction

Oral squamous cell carcinoma (OSCC) is one of the most common head and neck malignant tumors encountered globally, with high rates of incidence and mortality [1,2]. In recent decades, the number of cases in Asia has gradually increased [3]. Although the etiology of OSCC is reported to be related to smoking, alcohol consumption and chewing tobacco and betel nuts, the main risk factors are still unknown. Surgery combined with chemotherapy and radiotherapy has been widely used during the past four decades, with only a very slight improvement in patient survival [4–6]. The prognosis of patients with OSCC is still poor, with a 5-year survival rate of < 50% in most parts of the world [7]. These challenges encourage scientists to search for new risk factors and pathogenic mechanisms.

Since the first report of the relationship between gastric carcinogenesis and *Helicobacter pylori* published in the 1990s, studies investigating the relationship between bacteria and tumors have attracted increased attention [8]. Ample evidence suggests that bacterial infections are strongly associated with a variety of cancers, accounting for about 13% of the global incidence [9,10]. Most cancers, such as oral, prostate, breast, and ovarian types exhibit unique bacterial profiles and functional relationships [11–13]. The imbalance between oral bacteria and host can also contribute to oral and systemic diseases, as well as tumorigenesis [14]. However, the mechanism underlying the role of oral bacteria in OSCC occurrence and outcome remains to be explored.

Actually, inflammation has been considered as one of the dominant factors contributing to bacterial carcinogenesis [15]. Bacteria induce tumors by creating pro-inflammatory microenvironment or interfering with normal immune response [16–18]. The imbalance in microbial ecology is closely related to host inflammation and occurrence of various cancers, such as colon and gastric cancers [19,20]. Additionally, epidemiological studies have found that the ecological imbalance of saliva microbiome and the excessive release of pro-inflammatory cytokines contribute to OSCC via inflammation and related signaling pathways [21]. Lipopolysaccharide (LPS), a major cell-wall components of gram-negative bacteria, is a key exogenous virulence factors involved in oral bacterial metabolism [22]. LPS triggers strong immune responses and leads to severe infections, especially inducing inflammation and ferroptosis in periodontal tissue [23,24]. LPS alters the host gene expression profile to regulate the occurrence and progression of cancers [25]. Additionally, LPS is an activator of toll-like receptor 4 (TLR4), and the activation of downstream signaling pathways (including nuclear factors-κB (NF-κB) pathway and MAPK pathway) enhances the invasion and migration of cancer cells [26,27]. In addition, LPS and LPS-induced pro-inflammatory cytokines promote the expression of adhesion molecules on cancer and endothelial cells, thereby promoting the metastasis of cancer cells outside normal tissues [21]. A comparative analysis of bacterial composition of OSCC and normal tissues revealed that the proportion of *Fusobacterium nucleatum* and *Pseudomonas aeruginosa* was significantly increased in OSCC. Functional prediction revealed the enrichment of pro-inflammatory LPS in tumor tissue [28]. Thus, exploring the regulatory pattern of oral bacteria in OSCC provides insights into disease pathogenesis and identification of novel therapeutic targets.

In this study, we explored the potential mechanisms by which oral bacteria affect the occurrence and development of OSCC. Given that LPS is the most important bacterial virulence factor that could lead to the occurrence and development of various oral cancers, this study first combined GEO and GeneCards databases to screen LPS-related differentially expressed genes. Gene Ontology (GO) and Kyoto Encyclopedia of Genes and Genomes (KEGG) enrichment analysis were then conducted to explore the potential role of oral bacteria in the occurrence and outcome of OSCC. Subsequently, based on seven integrated algorithms, hub genes were screened out to investigate immune infiltration as well as the impact of oral bacteria on the occurrence, development, and treatment of OSCC. The study investigates the relationship between OSCC and oral bacteria provides a standard of reference for OSCC prevention and treatment.

## 2. Materials and methods

### 2.1. Acquisition and preprocessing of public data

The expression profiles of OSCC microarrays containing control samples and OSCC samples were acquired from the Gene Expression Omnibus (http://www.ncbi.nlm.nih.gov/geo/) database, including GSE37991, GSE74530 and GSE30784, which were then merged using the R package “inSilicoMerging” (version 1.17.0.1). The batch effect was eliminated via the “ComBat” method in R package “sva” (version 3.20.0).

### 2.2. Identification of LPS-related differentially expressed genes (LR-DEGs) in OSCC

R package “limma” (version 3.40.6) was employed to select differentially expressed genes (DEGs) in each OSCC dataset. Genes with P < 0.05 and |log fold change (FC)| ≥ 1 were considered as DEGs. GeneCards database was used to investigate genes related to bacterial LPS. Common genes of the two databases were identified as LR-DEGs.

### 2.3. Functional and pathway enrichment analysis of LR-DEGs

In order to explore the potential molecular mechanism of LPS affecting OSCC, DAVID (https://david.ncifcrf.gov/) was undertaken to conduct GO and KEGG enrichment analyses of the LR-DEGs. GO enrichment analysis contained biological processes (BP), molecular functions (MF) and cellular components (CC). The cut-off criteria were P < 0.05 and a false discovery rate (FDR) < 5%.

### 2.4. Protein-protein interaction network (PPI) construction, module analysis and identification of LPS-related hub genes (LRHGs)

The PPI network of LR-DEGs was constructed in STRING (http://www.string-db.org/). Both direct and indirect protein-protein functional interaction scores were predicted via this online database. The LR-DEGs with interaction combined score ≥ 0.4 were extracted for the construction of the PPI network. Cytoscape (version 3.9.1) was then utilized to visualize the network. Subsequently, module analysis was investigated via molecular complex detection (MCODE). Moreover, seven algorithms (including MCC, MNC, Degree, Closeness, Radial, EPC and stress) in CytoHubba were used to comprehensively identify LRHGs. As a result, hub genes overlapping in the seven algorithms were identified as LRHGs, and the co-expression network of LRHGs was illustrated using GeneMANIA (http://www.genemania.org/).

### 2.5. Specific evaluation and diagnostic validation of LRHGs

To determine the diagnostic specificity of 5 LRHGs in OSCC, the R package pROC (version 1.17.0.1) was used to perform receiver operating characteristic (ROC) curve analysis. The confidence interval was set as 95%. The area under the ROC curve (AUC) ≥ 0.7 was considered significant (Li et al., 2021).

### 2.6. Clinical samples and ethics Statement

To experimentally validate these LRHGs, five patients with OSCC who never received chemotherapy or radiotherapy were enrolled in this study. OSCC tissues and paired adjacent para-cancerous tissue located 5 cm away from the tumor margin were obtained and immediately frozen in liquid nitrogen for subsequent experiments [27]. All participants were informed of the experimental procedure and signed the informed consent form. The experimental samples were obtained in this study from November 25,2023 to January 24,2024. The study was approved by the Ethics Committee of Affiliated Hospital of North Sichuan Medical College2023ER433−1.

### 2.7. Validation of LRHGs by qRT-PCR, WB, ELISA and IF

The tissues were sufficiently grinded, as described in the instruction manual. RNA extraction kit (Accurate Biotechnology, China) was used to extract total RNA from the OSCC tissues and control tissues. Then, RT Mix Kit with gDNA Clean (Accurate Biotechnology, China) was utilized to synthesize DNA. The transcription of mRNA was detected using SYBR green (Baoguang Biotechnology, China). The relative mRNA expression level of LRHGs (*Cxcl8, Cxcl10*, **Il-1*β*, *Il-6*, and *Mmp9*) was normalized with beta-actin and determined via 2-ΔΔCt method. The primer sequences of the five LRHGs and beta-actin were displayed in Supplementary S1 Table.

For western blot (WB) analysis, total protein was extracted from tissues using RIPA lysis buffer, and protein concentrations were measured using a BCA protein assay kit (Beyotime, China). Equal amounts of protein were separated by SDS-PAGE and transferred to PVDF membranes (Millipore, USA). The membranes were blocked with 5% non-fat milk and incubated overnight at 4°C with primary antibodies specific to MMP9 (CST, America), and beta-actin (internal control). After incubation with HRP-conjugated secondary antibodies, protein bands were visualized using an enhanced chemiluminescence (ECL) detection system.

For ELISA, tissue samples were processed using an ELISA kit (Servicebio, China) according to the manufacturer’s protocol to quantify the protein levels of CXCL8, CXCL10, IL-1β, and IL-6.

For immunofluorescence (IF) staining, OSCC and control tissues were embedded in OCT compound and snap-frozen in liquid nitrogen. Frozen tissue sections (8 µm thick) were cut using a cryostat and mounted on glass slides. The sections were fixed in cold acetone for 10 minutes and air-dried. After blocking with 5% BSA for 1 hour at room temperature, the sections were incubated overnight at 4°C with primary antibodies specific to CXCL8, CXCL10, IL-1β, IL-6, and MMP9(antibody details are provided in Supplementary S2 Table). Following primary antibody incubation, the sections were washed with PBS and incubated with fluorophore-conjugated secondary antibodies for 1 hour at room temperature in the dark. Nuclei were counterstained with DAPI, and the slides were mounted with antifade mounting medium. Fluorescent images were captured using a fluorescence microscope.

### 2.8. Evaluation of immunocytes infiltration levels

To further explore the differences in immune characteristics between OSCC and control samples, Cell-type Identification by Estimating Relative Subsets of RNA Transcripts x (CIBERSORTx) was employed to evaluate the infiltration level of immunocytes between OSCC group and control group by transforming the gene expression matrix into the composition of infiltrating immunocyte. Immunocytes with P < 0.05 were considered as statistically significant and selected for subsequent correlation analysis with LRHGs.

### 2.9. Statistical analyses

The statistical analysis commenced with F-tests to assess data distribution normality. For data following a normal distribution, t tests were conducted. Conversely, the Mann‒Whitney test was employed for nonnormally distributed data. To guarantee result reliability and reproducibility, each experiment was performed a minimum of three times with biological replicates. The results are presented as means ± standard errors of the means (SEMs). Statistical evaluation was carried out using Prism 8.1 software (GraphPad Software, USA), with P values below 0.05 deemed statistically significant.

## 3. Results

### 3.1. Identification of LR-DEGs in OSCC

**Fig 1:**
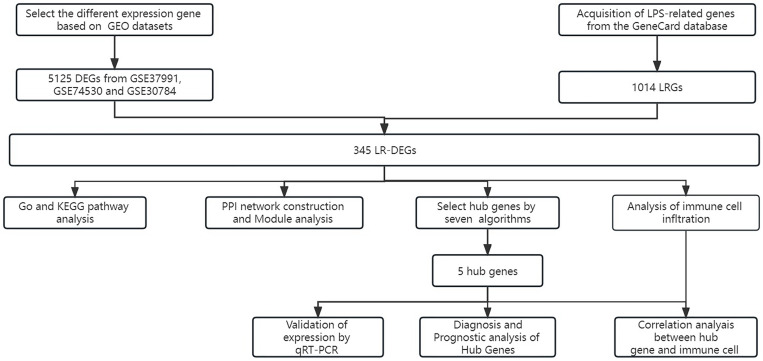
The flowchart outlining the study design.

**Fig 2:**
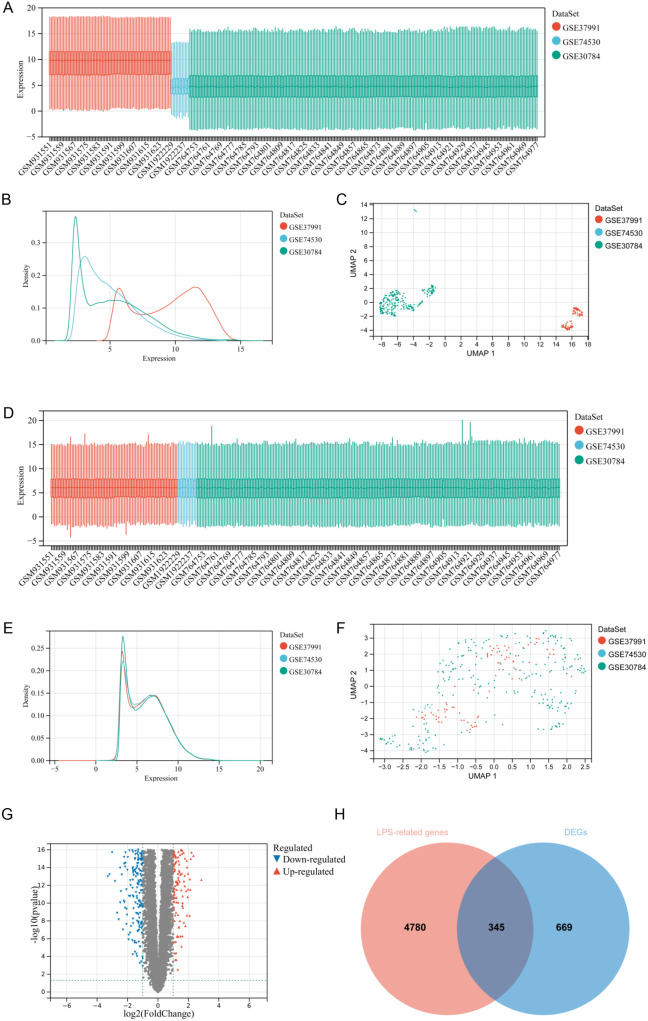
Data processing and LR-DEGs identification. **(A-C)** GEO microarray datasets GSE37991, GSE74530 and GSE30784 before data processing. **(D-F)** The GEO datasets microarray after data processing. **(G)** The volcano map of OSCC gene expression profile. Red indicated upregulated DEGs; blue indicated DEGs. **(H)** The Venn diagram illustrated LR-DEGs of OSCC.

The detailed flowchart of this study is illustrated in Fig 1. To identify novel genes in OSCC associated with LPS, we selected three OSCC datasets, including GSE37991, GSE74530, and GSE30784, and the detailed information of these datasets is presented in Table 1. The sequencing data of all patients in the three datasets were then processed following the procedure mentioned above. Compared with the data before processing, the results after processing demonstrated that the samples of each dataset were clustered and intertwined, which indicated favorable batch removal effects (Fig 2A-F). Subsequently, 5125 DEGs of OSCC were screened out (Fig 2G) and a total of 1014 LPS-related genes with a relevance score > 0.4 were obtained from GeneCards database. The DEGs overlapping LPS-related genes were identified as LR-DEGs. Totally, 345 common LR-DEGs were acquired (Fig 2H). The LR-DEGs were listed in Supplementary S3 Table.

**Table 1:** Detailed information about the three datasets used in the study.

ID	Author	Publish Year	Country	Platform	Sample type	OSCC samples	Normal samples
GSE30784	Chen C	2011	USA	GPL570	Human tissues	167	45
GSE37991	Lee CH	2013	China	GPL6883	Human tissues	40	40
GSE74530	Oghumu S	2017	USA	GPL570	Human tissues	6	6

### 3.2. Functional and pathway enrichment analysis of LR-DEGs

**Fig 3:**
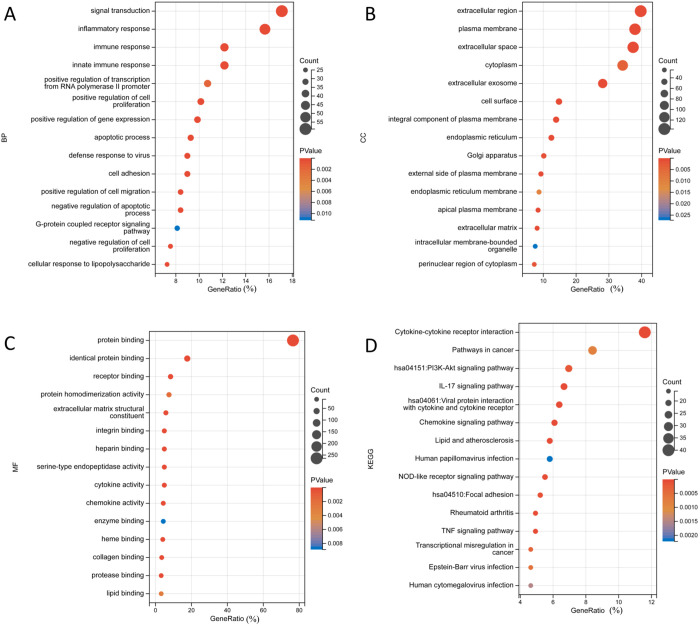
Functional and pathway enrichment analysis of LR-DEGs. (A) Top 10 BP terms. (B) Top 10 CC terms. (C) Top 10 MF terms. (D) Top 10 KEGG terms. P < 0.05 was considered statistically significant.

In order to explore the potential biological mechanisms of the 345 LR-DEGs, GO and KEGG pathway enrichment analyses were conducted. The results of GO analysis revealed that changes in BP of LR-DEGs included signal transduction, inflammatory response and immune response (Fig 3A). Changes in CC of LR-DEGs were mainly enriched in the basic cellular structure, such as plasma membrane, extracellular region and extracellular space (Fig 3B). Within the MF, the LR-DEGs were significantly enriched in protein binding, identical protein binding and receptor binding (Fig 3C). The KEGG pathway analysis indicated that the pathways involving cytokine-cytokine receptor interaction, pathways in cancer, PI3K-Akt and IL-17 signaling were significantly enriched (Fig 3D). Among them, IL-17 signaling pathway was reported to be the predominant pathway in host immune inflammatory response [28]. In addition to the biological mechanisms associated with cancer, these results also highlighted the critical role of LPS-induced inflammation in OSCC.

### 3.3. PPI network, module analysis and LRHG selection

**Fig 4:**
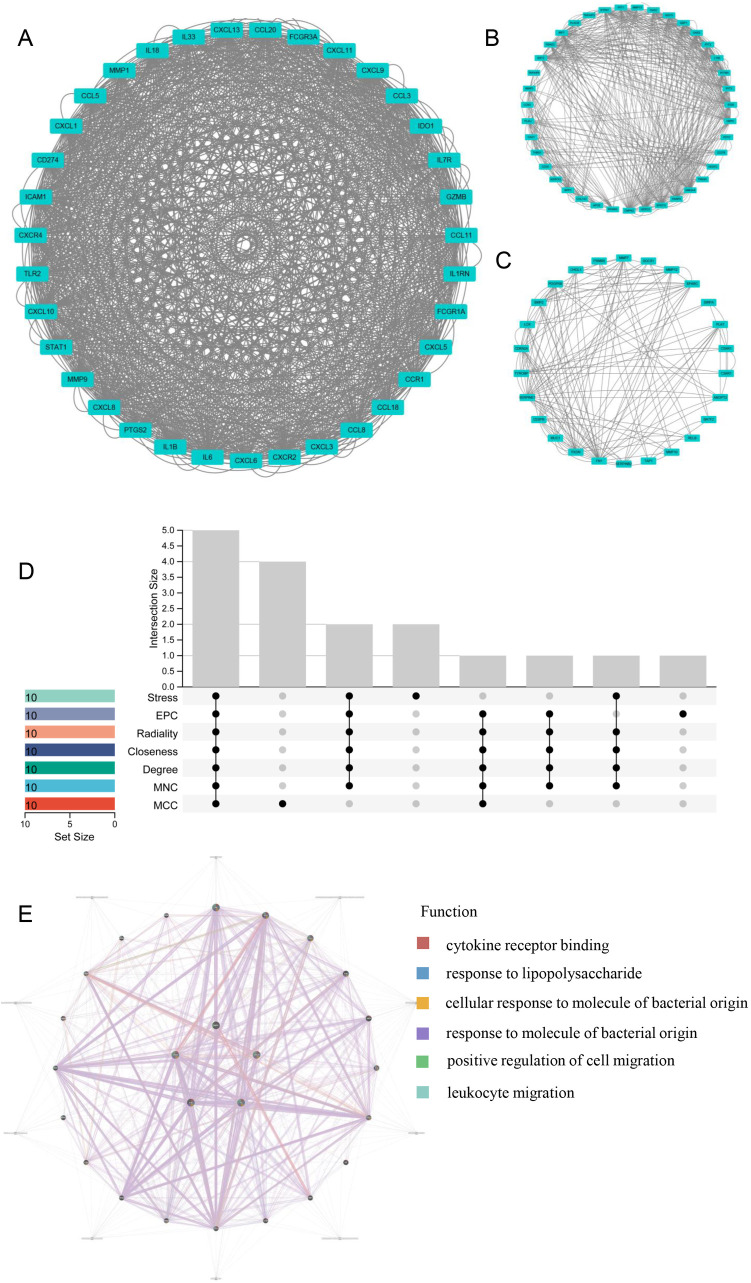
Significant modules of PPI network and LRHGs identification. **(A)** Significant module 1 (containing 34 nodes and 1038 edges). **(B)** Significant module 2 (containing 39 nodes and 504 edges). **(C)** Significant module 3 (containing 26 nodes and 146 edges). **(D)** The five overlapping LRHGs based on seven integrated algorithms. **(E)** Co-expression analysis of LRHGs.

PPI networks of LR-DEGs identified in the analysis above were constructed by STRING website, and the results were visualized in Cytoscape. Furthermore, three closely connected gene modules were identified as significant using a graph clustering algorithm known as MCODE. Briefly, module 1 comprised 35 nodes and 1070 edges (Fig 4A); module 2 contained 39 nodes and 504 edges (Fig 4B); and module 3 included 26 nodes and 146 edges (Fig 4C). The findings suggested that there were complex interaction regulatory networks between LR-DEGs and they might share potential molecular mechanisms of regulation in OSCC.

To make the results more accurate and credible, seven algorithms of CytoHubba were integratedly applied to identify common hub genes. By overlapping the results of seven algorithms, five LRHGs (including *Cxcl8, Cxcl10*, **Il-1*β*, *Il-6*, and *Mmp9*)) were selected (Fig 4D and Table 2). Subsequently, Table 3 presented the full names and partial functional annotation information of the five LRHGs. Besides, the co-expression networks and associated functions of LRHGs were determined using the GeneMANIA database (Fig 4E). The functional enrichment results of LRHGs suggested the mechanism of bacterial LPS and the response to immune regulation, which encouraged us to further explore the potential association between immune infiltration and OSCC, as well as the correlation between immunocytes and LRHGs.

**Table 2:** The hub genes were obtained by seven algorithms respectively.

algorithms	hub genes									
MCC	IL6	IL1B	CXCL8	CXCL10	MMP9	CCL3	CXCL1	CXCL9	ICAM1	CCL20
MNC	IL6	IL1B	CXCL8	CXCL10	MMP9	FN1	PTGS2	STAT1	ICAM1	CCL5
Degree	IL6	IL1B	CXCL8	CXCL10	MMP9	FN1	PTGS2	STAT1	ICAM1	CCL5
Closeness	IL6	IL1B	CXCL8	CXCL10	MMP9	FN1	PTGS2	STAT1	ICAM1	CCL5
Radiality	IL6	IL1B	CXCL8	CXCL10	MMP9	FN1	PTGS2	STAT1	ICAM1	CCL5
EPC	IL6	IL1B	CXCL8	CXCL10	MMP9	FN1	CXCR4	STAT1	ICAM1	CCL5
Stress	IL6	IL1B	CXCL8	CXCL10	MMP9	FN1	PTGS2	STAT1	PPARG	LTF

**Table 3:** Detailed information of the 5 LRHGs.

Gene	Full Name	Function
CXCL8	C-X-C motif chemokine ligand 8	It functions as a chemotactic factor by guiding the neutrophils to the site of infection.
CXCL10	C-X-C motif chemokine ligand 10	Binding of CLCL10 protein to CXCR3 can stimulate migration of monocytes, natural killer and T-cell, and modulation of adhesion molecule expression.
IL-1β	interleukin 1 beta	This cytokine is an important mediator of the inflammatory response, and is involved in a variety of cellular activities, including cell proliferation, differentiation, and apoptosis.
IL-6	interleukin 6	The protein is primarily produced at sites of acute and chronic inflammation, where it is secreted into the serum and induces a transcriptional inflammatory response through interleukin 6 receptor, alpha.
MMP9	matrix metallopeptidase 9	It involved in the breakdown of extracellular matrix in normal physiological processes and disease processes

### 3.4. Evaluation of the diagnostic role of LRHGs in OSCC

**Fig 5:**
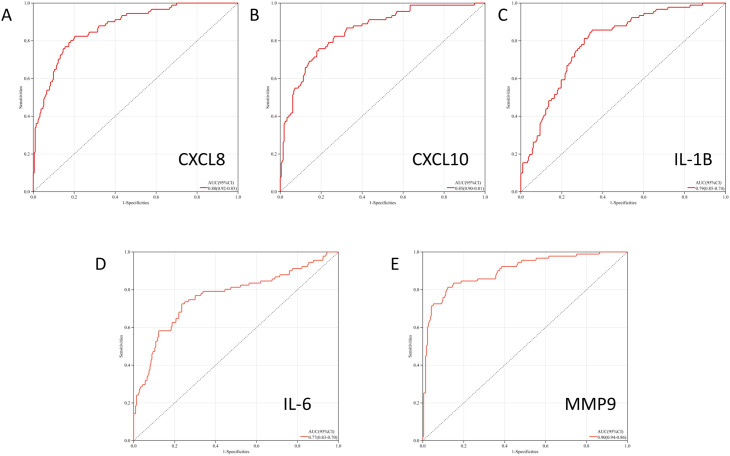
The ROC analysis and AUC of CXCL8, CXCL10, IL1β, IL6 and MMP9, respectively.

The ROC curve analysis was undertaken to evaluate the diagnostic efficacy of LRHGs in OSCC. Based on the mRNA expression profile of sequencing data in GEO database, the AUC values of *Cxcl8, Cxcl10*, **Il-1*β*, *Il-6*, and *Mmp9* were 0.88, 0.85, 0.79, 0.77 and 0.90, respectively (Fig 5, P < 0.05). These data demonstrated that the five LRHGs represented promising diagnostic biomarkers of OSCC.

### 3.5. Experimental validation of LRHGs by qRT-PCR, WB, ELISA and IF

**Fig 6:**
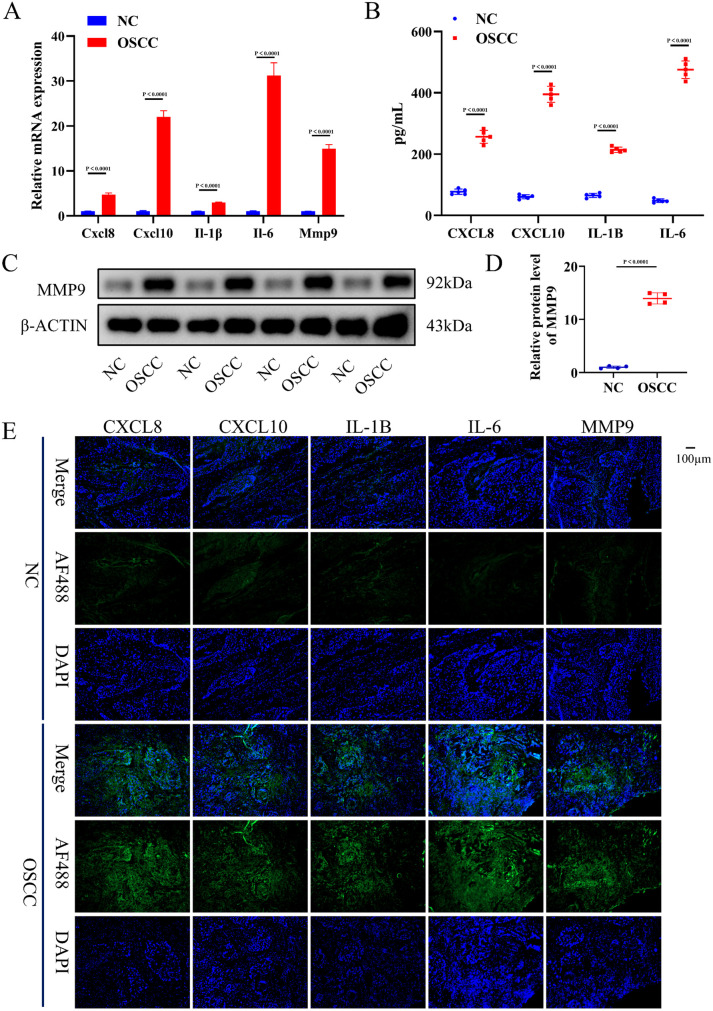
Experimental validation of CXCL8, CXCL10, IL1β, IL6 and MMP9, respectively. (A) qRT-PCR analysis of differential mRNA expression of LRHGs between the OSCC and control groups. **(B)** ELISA was performed to assess the protein expression levels of CXCL8, CXCL10, IL-1B, and IL-6 between the OSCC and control groups. **(C-D)** Western blot analysis of MMP9 protein expression between the OSCC and control groups, with quantitative analysis conducted using Image-J. **(F)** Immunofluorescence detection and localization of LRHGs protein in the OSCC and control groups. P < 0.05, P < 0.01, P < 0.001.

The expression of LPS-related hub genes (LRHGs) was comprehensively analyzed in oral squamous cell carcinoma (OSCC) tissues and corresponding normal controls at both the mRNA and protein levels. Quantitative real-time PCR (qRT-PCR) revealed a statistically significant upregulation of all LRHGs, including *Cxcl8, Cxcl10*, **Il-1*β*, *Il-6*, and *Mmp9*, in OSCC tissues compared to controls (Fig 6A, P < 0.05). At the protein level, both enzyme-linked immunosorbent assay (ELISA) and Western blot (WB) analyses corroborated these findings, demonstrating a significant increase in the protein expression of CXCL8, CXCL10, IL-1β, IL-6 and MMP9 in OSCC tissues relative to normal controls (Fig 6B-D, P < 0.05). Additionally, immunofluorescence (IF) staining exhibited markedly enhanced fluorescence intensity for CXCL8, CXCL10, IL-1β, IL-6 and MMP9 in OSCC tissues, further validating the elevated protein expression observed in both ELISA and WB experiments (Fig 6E). These data collectively suggest that the upregulation of LRHGs is closely linked to OSCC progression, likely mediated through the pro-inflammatory effects of lipopolysaccharide (LPS), implicating LRHGs as potential contributors to OSCC development via LPS-induced inflammatory pathways.

### 3.6. LRHG expression correlates with immunocytes infiltration in OSCC

**Fig 7:**
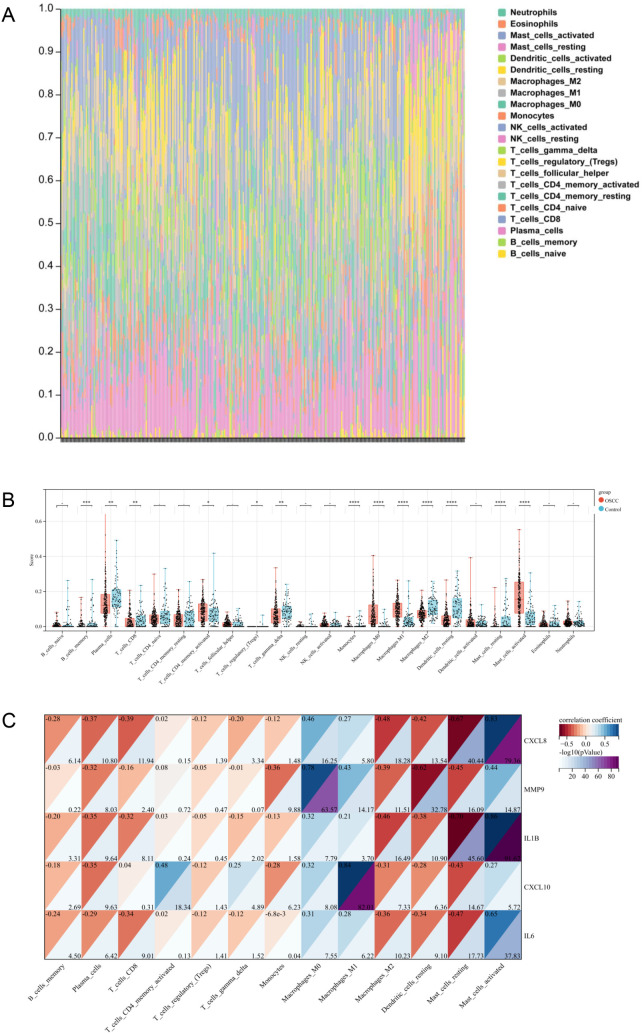
Different immunocyte infiltration in OSCC and their association with LRHGs. **(A)** Immunocyte proportions in OSCC and control samples. **(B)** Evaluation of immunocytes infiltration levels between OSCC group and control group. Control samples were shown in blue and OSCC samples in red. **(C)** Correlation analysis between LRHGs and immunocytes. *P < 0.05, **P < 0.01, ***P < 0.001.

Fig 7A illustrated the infiltration levels of immunocytes expression in each sample. Totally, 22 immunocytes subtypes were investigated. All immunocytes except for B cell naive, T cell CD4 naive, resting NK cells, resting CD4 memory T cells, follicular helper T cells, activated NK cells, activated dendritic cells, eosinophils and neutrophils showed significantly different levels of infiltration between OSCC group and control group (Fig 7B). These immunocytes with significant differences were selected for subsequent analysis. Notably, the four types of immunocytes (including active CD4 memory T cells, M0 macrophages, M1 macrophages and active mast cells) demonstrated higher infiltration levels of immunocytes in OSCC than in control group (Fig 7B). The distinct immunocytes infiltration pattern between OSCC group and control group indicated the vital role of immune regulation in the pathogenesis of OSCC.

In order to explore the potential relationship between LPS-related genes and immune mechanisms in OSCC, we analyzed the correlation between the 13 immunocytes and the LRHGs. The heatmap illustrated that the *Cxcl8* expression was strongly positively correlated with activated mast cells (r = 0.83, Fig 7C). *Mmp9* showed strong correlation with M0 macrophages (r = 0.78, Fig 7C), while Il-1β was strongly correlated with mast cells (| r | ≥ 0.70, Fig 7C). Cxcl10 exhibited the strongest correlation with M1 macrophages (r = 0.84, Fig 7C). The results of correlation analysis suggested that LRHGs might regulate the infiltration levels of immunocytes into OSCC tumor tissues. Overall, these findings indicated that bacterial LPS was closely associated with immune response in patients with OSCC.

## 4. Discussion

OSCC is one of the most invasive epithelial malignancies that adversely affect patients’ appearance and quality of life [29,30]. The incidence of OSCC is notably high, especially in Asian countries [31]. However, the pathogenic mechanism is still unclear. The role of bacteria in tumor development and progression has attracted increased attention, and more than 15% of cases are related to specific pathogenic bacteria [32]. The types of oral bacteria and their biofunction in OSCC are diverse and contribute to various stages of tumor occurrence, development, metastasis, and immune response. Therefore, to further explore the potential relationship between OSCC and bacteria, bioinformatics and experimental validation were applied to explore the bacterial regulatory mechanisms in OSCC.

Among the multitudinous pathogenic factors of bacteria, LPS is a key component of the cell wall of most gram-negative bacteria and induces cytotoxicity [33]. It is also the main bacterial constituent inducing host immune response and typically plays an important role in bacterial infections. LPS is released into the surrounding medium after cell division, detachment of outer membrane vesicles, or bacterial cell death. It induces pro-inflammatory immune response in host, resulting in sepsis and even death [34]. Chronic infection increases the risk of malignant tumors [35]. It has been reported that multiple pro-inflammatory cytokines in the tumor microenvironment promoted tumor growth rather than tumor inhibition [36,37].

In this study, we identified DEGs of OSCC using the GEO database, and combined with the LPS related genes of the GeneCards database to obtain LR-DEGs of OSCC. Then, a combination of experimental validation, functional enrichment, protein interaction co-expression networks, hub gene selection, and immune infiltration evaluation was employed to investigate the molecular mechanisms of the regulation of oral bacterial on OSCC. Finally, we identified 345 LR-DEGs with significant differences from 3 GEO datasets of OSCC. GO functional enrichment analysis revealed that DEGs were mainly enriched in inflammation related biological processes involving signal transduction, inflammatory response, and immune response. Besides, cell composition enrichment analysis results (such as extracellular region and plasma membrane) and molecular functions enrichment analysis results (such as protein binding and receiver binding) were also screened out. Importantly, KEGG pathway enrichment analysis results included cytokine-cytokine receptor interaction, pathways in cancer, the PI3K-Akt signaling pathway and IL-17 signaling, which were mainly enriched and strongly related to inflammatory response. Collectively, these above results indicated that bacterial inflammation mediated by LPS-related genes facilitates the development of OSCC.

This study ultimately screened out five potential OSCC diagnostic biomarkers with high sensitivity and specificity via seven integrated algorithms, including *Cxcl8, Cxcl10*, **Il-1*β*, *Il-6*, and *Mmp9*. Cxcl8 is a typical chemokine belonging to the CXC family, responsible for recruiting and activating neutrophils and granulocytes to the inflammatory sites [38]. It is almost undetectable under physiological conditions, but the levels of CXCL8 are rapidly induced by pro-inflammatory cytokines [39]. Changes in the CXCL8 signaling pathway inhibited cellular apoptosis and promoted multidrug resistance, thereby reducing the sensitivity of cancer cells to chemotherapy [40,41]. Upregulation of CXCL8 expression exhibited an increased risk of cancer and poor prognosis of normal patients [42]. CXCL10, also known as human interferon-inducible protein 10 (IP-10), is an intrinsic cytokine involved in regulating adaptive immune response and plays a vital role in combating infections and malignant tumors [43]. Overexpression of CXCL10 in HeLa cells could activate TP53-mediated apoptosis [44]. Previous studies reported that CXCL10 had a negative correlation with the survival time of the patients, suggesting it could be a poor prognostic indicator of pancreatic adenocarcinoma [45].

IL-1β is an important pro-inflammatory cytokine in the immune inflammatory response and a predominant mediator of pyroptosis and apoptosis in cells [46]. Increased expression and secretion of IL-1β by cancer stem cells might activate the autocrine signaling loop by stimulating signaling pathways (such as NF-κB and cAMP-response element binding protein pathways) and promoted epithelial mesenchymal transformation, cell invasion and metastasis [47]. Besides, the overexpression of IL-1β shown poor prognosis of patients with oral and nasopharyngeal squamous cell carcinoma [48]. IL-6 is a multifunctional cytokine mainly produced in the tumor microenvironment and is a key factor driving tumor growth and metastasis [49]. It was reported that IL-6 promoted distant metastasis of tumors by promoting epithelial mesenchymal transformation of cancer cells [50]. IL-6 promoted the proliferation of head and neck carcinoma cells in vitro. Moreover, inhibiting IL-6 signaling significantly reduced the growth rate of cancer cells [51]. MMP9 is a zinc-dependent proteolytic enzyme that degrades extracellular matrix (ECM) collagen, which can facilitate tumor invasion and metastasis [52]. MMP9 not only plays a role in angiogenesis, metastasis, and cancer invasion, but also contributes to the survival and spread of cancer cells. Many clinical and experimental studies reported that the expression of MMP9 increased with cancer progression [53]. The overexpression of MMP9 was associated with poor prognosis of patients diagnosed with oral or breast cancer [54,55].

In this study, all proteins encoded by LRHGs were closely related to inflammation of cancer, suggesting that bacterial LPS could regulate the progression of OSCC through inflammation. The results of qRT-PCR, WB, ELISA and IF demonstrated that LRHG transcriptions were upregulated in OSCC, compared with control group. The ROC curve analysis illustrated that the five LRHGs had favorable diagnostic value for OSCC. LPS is an important factor associated with enhanced host inflammatory response. It activated the intracellular signal transduction mechanism related to NF-κB and the cell surface receptor TLR4, and further upregulated the downstream pro-inflammatory factors, culminating in the aggravation of cancer inflammation [56]. Following LPS stimulation, P65, the downstream NF-κB subunit, was phosphorylated after nuclear translocation, inducing other pro-inflammatory signaling factors, such as interleukins IL-1β, IL-6 and tumor necrosis factor-α (TNF-α). Induction of additional chemokines and pro-inflammatory factors further exacerbated the inflammatory reactions and the destruction of cancer tissue integrity [57]. The upregulation of pro-inflammatory cytokines, including CXCL8, CXCL10, IL-1β, IL-6 and MMP9 was associated with the progression of host bacterial infection. Therefore, the study findings suggested that inflammation in cancer wass a key mechanism linking bacterial LPS and OSCC, and LPS increased the risk and adverse outcomes of OSCC.

Immunotherapy is currently an effective strategy for treating advanced cancers. However, the effectiveness of immunotherapy depends largely on the patients’ tumor microenvironment (TME) [58]. TME refers to the presence of non-cancer cells and components in tumors, including the molecules synthesized and released, which can be used to predict disease outcomes and evaluate immunotherapeutic effects [59]. OSCC has characteristic TME, and changes in OSCC TME balance due to altered immunocytes populations, immune checkpoints and tumor microenvironment factors in cancers are likely to facilitate the escape of cancer cells from immune surveillance [60]. t is well known that immunocytes infiltration is a key index of the prognosis of OSCC and other cancers [61]. Our results demonstrated that LRHGs promoted immunocytes infiltration and eventually contributed to the morbidity and metastasis of OSCC. Therefore, bacterial LPS was considered to be associated with the regulation of OSCC immune response and represents a promising target for immunotherapy in patients with OSCC.

The role of oral bacteria in OSCC pathogenesis has been established initially. However, the dominant pathogenic mechanism is still unknown. Our study investigated the potential relationship between oral bacteria and OSCC based on bioinformation analysis combined with experimental validation. In ongoing work, we plan to analyze NGS datasets to provide a deeper understanding and explore additional LPS-related pathways. Collectively, LPS was found to be a potential etiological factor and a promising immunotherapeutic target of OSCC. Further experimental evidence is needed to validate the diagnostic role of bacteria in OSCC, as well as its prevention and immunotherapeutic effects.

## 5. Conclusion

The findings indicated that LPS was a key component regulating the interaction between oral bacteria and OSCC. The results of qRT-PCR revealed that five LRHGs were upregulated in OSCC. The validation of LRHGs and ROC analysis suggested that the five LRHGs (*Cxcl8, Cxcl10*, **Il-1*β*, *Il-6*, and *Mmp9*) were potential diagnostic molecular biomarkers of OSCC. The results of immune infiltration analysis illustrated that immunotherapy targeting LRHGs might improve the prognosis of patients with OSCC. In general, our study explored a complex regulatory mechanism involving oral bacteria, which affected the progression and outcome of OSCC via LPS and inflammation. The findings broadened the understanding of the potential molecular mechanisms of OSCC and provided novel insights into the development of OSCC treatment.

## Supporting information

S1 Table Primer sequence for qRT-PCR of human samples. (DOCX)

S2 Table Primary antibodies used in this study. (DOCX)

S3 Table LR-DEGS. (XLSX)
