# Supplementary material for: Bioinformatics analysis and experimental validation of the potential relationship between bacterial lipopolysaccharide and oral squamous cell carcinoma
Source: PLoS One. 2025 Aug 21;20(8):e0329231. doi: 10.1371/journal.pone.0329231 (PMC12370139; doi:10.1371/journal.pone.0329231)
Supplement: S1 Table — (DOCX) [file pone.0329231.s001.docx]

Table S1. Primer sequence for qRT-PCR of human samples

| Genes | Upstream (5’–3’) | Downstream (5’–3’) |
| --- | --- | --- |
| *β-actin* | TGGCACCCAGCACAATGAA | CTAAGTCATAGTCCGCCTAGAAGCA |
| *Mmp9* | ACGCACGACGTCTTCCAGTA | CCACCTGGTTCAACTCACTCC |
| *Cxcl-10* | TGCTGCCTTATCTTTCTGACTCT | GGACAAAATTGGCTTGCAGGAAT |
| *Il-8* | TTGGCAGCCTTCCTGATTTC | AAAGTGCTCTGTTGTAGTGGAAAG |
| *Il-1β* | TCTGTACCTGTCCTGCGTGT | ACTGGGCAGACTCAAATTCC |
| *Il-6* | GAGTAGTGAGGAACAAGCCAGAG | GGTCAGGGGTGGTTATTGC |
