# Supplementary material for: Bioinformatics analysis and experimental validation of the potential relationship between bacterial lipopolysaccharide and oral squamous cell carcinoma
Source: PLoS One. 2025 Aug 21;20(8):e0329231. doi: 10.1371/journal.pone.0329231 (PMC12370139; doi:10.1371/journal.pone.0329231)
Supplement: S2 Table — (DOCX) [file pone.0329231.s002.docx]

Table S2. Primary Antibodies Used in This Study

| Antibody | Vendor | Catolog/Institution | Clonality |
| --- | --- | --- | --- |
| β-ACTIN | Proteintech | 81115-1-RR | recombinant |
| CXCL8 | Santa | sc-8427 | monoclonal |
| CXCL10 | Santa | sc-8438 | monoclonal |
| IL-6 | Santa | sc-130326 | monoclonal |
| IL-1B | Santa | sc-52013 | monoclonal |
| MMP9 | Santa | sc-21733 | monoclonal |
